# Experiments in Therapeutics

**Published:** 1888-10

**Authors:** 


### HOMŒOPATHIC PHYSICIAN,

A MONTHLY JOURNAL OF

HOMŒOPATHIC MATERIA MEDICA AND CLINICAL MEDICINE.

"If our school ever give up the strict inductive method of Hahnemann, we are lost, and deserve only to be mentioned as a caricature in the history of medicine."—CONSTANTINE HERING.

Vol. VIII. OCTOBER, 1888. No. 10.

### EXPERIMENTS IN THERAPEUTICS.

Experiment is defined as "an operation to discover or prove some truth." Experiments in therapeutics are therefore operations to discover or prove some truth in therapeutics. When, therefore, we read of homœopathic physicians "experimenting" with some drug, it is to be presumed they are trying to prove some therapeutic truth as regards the action of homœopathic medicines. Hahnemann has ably shown us how to make pure, inductive experiments with drugs; his demonstration of pure drug experiment is so forcible and so true that even the old school of medicine has adopted it to a great extent. It would seem entirely unnecessary, at this late day, to attempt any demonstration of the truth of Hahnemann's teachings on this subject of drug experiment. He tells us so plainly that all drugs must be proven on healthy persons before they can be used to cure the sick; in this way we know *in advance of any clinical experiment* just what each proven drug is able to cure. This is the only pure experiment in regard to drug action of which we have any knowledge, and all other methods of drug experimentation are simply empirical, uncertain, and bring no one any useful information.

The truth of Hahnemann's teaching on this subject of drug proving has been so completely proven by the united experience of both the homœopathic and allopathic schools of medicine that it would be simply insulting the intelligence of medical men to attempt any further demonstration of it. These facts being acknowledged, is it not surprising that one should read from time to time of any physician's experimenting with an unproven remedy to see if it be not "good for" some disease? To use an unproven remedy is committing one sin; to prescribe any drug (proven or unproven) for a disease, *per se*, is committing another sin; in either case the so-called experimenter (being one who risks success on chance, gambler would be the better name) is totally disregarding the two basic facts upon which chiefly rests the system of homœopathic medical philosophy. These two facts are the proving of drugs upon the healthy and prescribing them for symptoms only.

In a recent volume of transactions of a certain society, we read of one physician who spoke highly of a remedy for "gangrene;" of others who discussed the value of a remedy, said to be good for "painful parturition." Many others have remedies for "chills," or for "diarrhœa," etc. Now, all these so-called experiments are entirely wrong, alike to the physician and to the profession at large. The physician who tries them learns nothing useful, and is in danger of getting into a careless routine method of prescribing. The discussion or the publication of any such experiments is very apt to lead astray some of the younger or less skillful physicians.

There are two methods of experimenting in therapeutics; the one is the old, fallacious method, tried in the old school since the days of Hippocrates, the clinical experiment; the other is the new, the true and the successful one, the pathogenetic, introduced by Hahnemann. It is to be hoped the homœopathic school is not going to retrograde and to return to the old empirical methods of Hippocrates!

There is another tendency, prevalent with some of the truest homœopathists, which is to be deprecated. This is a leniency exercised toward empirical experiments or prescriptions in which the higher potencies are used. Very many physicians are in this way disposed to credit and approve of any experiment if the medicine used be given in a CM or similar potency; whereas, had the drug been administered in a tincture, it is probable they would consider the practice "mongrel."

Hahnemann experimented with drugs in such a true fashion as to give the medical world its first and its only true materia medica. Every drug so experimented with added facts, useful, reliable facts, to the record of medical sciences. But can any one recall a single useful fact added by the clinical method of Hippocrates?

Let us not forget this parting warning of Constantine Hering, "If our school ever give up the strict inductive method of Hahnemann, we are lost, and deserve only to be mentioned as a caricature in the history of medicine."

